# Barriers to Discharge for Nursing Home Residents With Serious Mental Illness

**DOI:** 10.1001/jamanetworkopen.2025.34685

**Published:** 2025-09-30

**Authors:** Charity M. Hoffman, Hannah C. Ratliff, Sarah L. Krein, Kierstdea Petzold, Kathleen T. Unroe, Kali S. Thomas, Molly Turnwald, Erika Ratliff, Terrence Gatton, Donovan T. Maust

**Affiliations:** 1 Department of Psychiatry, University of Michigan, Ann Arbor; 2 Department of Internal Medicine, University of Michigan, Ann Arbor; 3 Center for Clinical Management Research, VA Ann Arbor Healthcare System, Ann Arbor, Michigan; 4 Institute for Healthcare Policy and Innovation, University of Michigan, Ann Arbor; 5 Department of Medicine, Geriatrics, Indiana University, Indianapolis; 6 Johns Hopkins School of Nursing, Baltimore, Maryland

## Abstract

**Importance:**

Nursing homes deliver rehabilitative and long-term care for people with serious medical illnesses, functional impairment, and/or cognitive impairment. The population of nursing home residents with serious mental illness (ie, bipolar disorder, schizophrenia, other psychotic disorders) has grown substantially. Other than prisons and jails, nursing homes are the largest institutional care setting for adults (aged ≥18 years) with serious mental illness. Nursing home residents with serious mental illness become long-term residents despite less functional impairment, which may be a function of difficulty discharging these individuals to the community.

**Objective:**

To examine barriers associated with discharging nursing home residents with serious mental illness back to the community from the perspective of frontline staff and administrative leaders.

**Design, Setting, and Participants:**

This qualitative study included semistructured interviews conducted by phone and videoconferencing with staff members at nursing homes across the US between August 29, 2024, and January 9, 2025. Nursing homes that deliver care to patients with serious mental illness were identified using LTCFocus.

**Main Outcomes and Measures:**

Themes and subthemes that represented barriers to discharge were analyzed from interview notes and transcripts using a rapid qualitative analysis approach.

**Results:**

Interviews were conducted with 15 staff members (mean [SD] age, 44.7 [12.6] years; 13 women [86.7%]) from 8 nursing homes. Staff roles included social services directors (8 participants [53.3%]), social workers or related roles (3 participants [20.0%]), and administrators or other roles (eg, executive director, memory care coordinator, behavioral unit manager of a lock unit) (4 participants [26.7%]). Participants identified multiple barriers to discharge at individual (eg, behavioral issues, medication compliance, withholding information, refusal to leave, comorbidities), relational (eg, lack of family support, strain on professional relationships), and structural (eg, limitations of insurance coverage, barriers to facility admissions, lack of substance and psychiatric treatment, waiting lists, transportation) levels, which together limited pathways to community discharge for residents with serious mental illness.

**Conclusions and Relevance:**

This qualitative study found that multiple intersecting barriers are associated with discharging nursing home residents with serious mental illness back to the community, which may lead to clinically inappropriate long-term stays for those who could theoretically have been in a lower level of care setting. These findings suggest that enabling successful discharge to the community for nursing home residents with serious mental illness may require collaborative strategies that address individual, relational, and structural factors.

## Key Points

**Question:**

What barriers are associated with discharging residents with serious mental illness, such as bipolar disorder, schizophrenia, or psychotic disorders, from nursing homes?

**Findings:**

In this qualitative study including 15 staff members from 8 nursing homes across the US, participants reported multifaceted barriers to discharge for residents with serious mental illness from individual characteristics to relational and structural issues. Barriers included resident behaviors due to serious mental illness, limited access to psychiatric treatment, lack of robust social support networks, limitations in insurance coverage, and waiting lists for limited alternative housing options.

**Meaning:**

These findings suggest that facilitating discharge from nursing homes to the community for residents with serious mental illness may require complex strategies that consider individual, relational, and structural interventions, ranging from education for staff and families to tiered supportive residential housing options.

## Teaser

This qualitative study examines barriers associated with discharging nursing home residents with serious mental illness into the community from the perspective of frontline staff and administrators.

## Introduction

The proportion of nursing home residents with serious mental illness, defined here as bipolar disorder, schizophrenia, or other psychotic disorders, nearly doubled over just 10 years, from 10.5% in 2007 to 18.6% by 2017.^1^ Nursing homes are second only to prisons and jails as the largest institutional care setting for adults (aged ≥18 years) with serious mental illness,^2,3^ despite the federally mandated Preadmission Screening and Resident Review program and the 1999 US Supreme Court Olmstead decision. The Preadmission Screening and Resident Review program requires states to screen individuals with serious mental illness (level I) prior to nursing home admission to determine appropriate placement and services (level II). The Olmstead decision ruled that nursing home settings were discriminatory for individuals who would otherwise prefer to live in the community.^4,5^ The nearly 150 000 individuals with serious mental illness residing in nursing homes comprise a population that equals the entire supply of inpatient psychiatric beds in the US.^1,6^

Most patients enter a nursing home for rehabilitation or other medical services following hospitalization. Ideally, when nursing home care is no longer needed, residents are discharged. However, some individuals become long-stay (ie, >100 days) residents,^3^ with the nursing home essentially becoming their home. Long-stay residence occurs disproportionately for individuals with serious mental illness, despite their being more likely to have low care needs, such as not needing the functional, clinical, or rehabilitation services that typically lead to nursing home residency.^7^ Co-occurring substance use disorders (eg, alcohol or opioid use disorder), which are more prevalent among individuals with serious mental illness,^8^ may further complicate care and discharge planning given the complexity of services needed upon discharge.^9^ The higher likelihood of individuals with serious mental illness becoming long-stay residents despite low care needs suggests that there may be barriers to planning appropriate discharge to the community.

While the number of individuals with serious mental illness living in nursing homes continues to grow, we know little about this population, including barriers to discharge, which limits society’s ability to realize the vision of the Olmstead decision and support these individuals in the community. To gain insights into this issue, we conducted interviews with frontline staff and administrators from nursing homes across the US to identify barriers associated with discharge of individuals with serious mental illness after receiving care in a nursing home.

## Methods

This qualitative study involved semistructured interviews conducted between August 29, 2024, and January 9, 2025, of staff at nursing homes in the US that serve residents with serious mental illness. The study was part of a larger project on the management of care for residents with serious mental illness in the nursing home setting. This research was determined exempt from full review by the University of Michigan Institutional Review Board because it poses no more than minimal risk to human participants and involves no intervention. The research was also exempted from documentation of consent, though we provided a verbal summary of our research project and goals to participants and obtained verbal consent. The study followed the Consolidated Criteria for Reporting Qualitative Research (COREQ) reporting guideline.^10^

We identified nursing homes that deliver care to patients with serious mental illness using LTCFocus, a data source sponsored by the National Institute on Aging available through a cooperative agreement with the Brown University School of Public Health.^11^ To ensure geographic and performance-related diversity, we identified facilities in the same zip code with similar proportions of residents with serious mental illness using Nursing Home Compare to identify facility pairs matched on higher (4-star or 5-star) and lower (1-star or 2-star) quality.^12^ We identified 74 matched pairs from approximately 14 000 nursing homes in the US. We sent introductory letters to facilities, followed by phone calls from study team clinicians (K.T.U. and D.T.M.) to nursing home administrators requesting facility participation and specifically, assistance connecting with staff involved in discharge planning so that we could directly invite them to participate in interviews.

### Data Collection

We conducted semistructured interviews of approximately 1 hour by videoconferencing or telephone. Interviews were conducted by a lead interviewer (C.M.H., a sociologist and social worker by training), with a second interviewer serving as notetaker (H.C.R., M.T.,E.R., or T.G.). The interview guide (eMethods in Supplement 1), constructed by the interdisciplinary study team, included questions about discharge planning procedures, unique considerations and barriers for residents with serious mental illness, and elements of a successful discharge. We also collected self-described basic demographic information (ie, age, race and ethnicity, gender, job role) to better understand the personal characteristics that might inform participants’ experiences. Participants received a $100 incentive payment for participating.

Interviews were recorded and transcribed verbatim; 2 participants consented only to note-taking because of employer policy. Interviews were conducted with staff at facilities across each of the 4 US census regions (Northeast, Midwest, South, and West) until responses became predictable, with no new concepts emerging (ie, achieving saturation).^13^

### Data Analysis

Transcripts were edited for accuracy and used to cross check preliminary notes. We used a rapid qualitative analysis method, summarizing interview notes into a template organized by the following key topics^14,15^: overview of discharge planning, barriers and resources for discharging residents with serious mental illness, family involvement, examples of successful and unsuccessful discharges, and other (ie, findings outside of key interest areas). Five authors (C.M.H., H.C.R., M.T, E.R., and T.G.) reviewed the summaries to identify initial themes, with data integrated into a matrix and further assessed to identify additional emergent themes and subthemes. Author C.M.H. identified initial concepts and grouped similar concepts into themes and subthemes, using a framework of structuration theory from sociology,^16^ with themes further refined through discussion with the interdisciplinary study team.

## Results

Across 8 participating facilities (Table 1), we completed interviews with 15 staff (mean [SD] age, 44.7 [12.6] years; 2 self-identified as men [13.3%] and 13 as women [86.7%]; 1 self-identified as Asian (6.7%), 5 as Black or African American (33.3%), 1 as Hispanic (6.7%), and 8 as White (53.3%) race and ethnicity) (Table 2). Staff roles included social service directors (8 participants [53.3%]), social workers or related roles (3 participants [20.0%]), and administrators or other roles (ie, executive director, memory care coordinator, behavioral unit manager of a locked unit) (4 participants [26.7%]).

**Table 1.** Facility Characteristics

Facility	Census region	No. of certified beds^a^	County population^a^^,^^b^	County rurality^a^^,^^b^
1	Northeast	101-200	Low	High
2	Northeast	>200	High	Low
3	Midwest	>200	High	Low
4	Midwest	101-200	Low	Medium
5	Midwest	0-100	Low	Medium
6	South	101-200	Low	High
7	West	101-200	High	Medium
8	West	0-100	High	Low

^a^ The number of certified beds was derived from LTCFocus, and county population and rurality were determined using US Census data.

^b^ Tertiles for participating facilities were assigned based on overall characteristics for nursing homes in the US. At the county level, fewer than 157 000 people was considered as low, 157 000 to 830 000 people as medium, and more than 830 000 people as high population. County rurality refers to the percentage of the county’s population who lives in a census-defined rural area, with less than 3.8% considered as low, 3.9% to 26.5% as medium, and more than 26.5% as high population.

**Table 2.** Participant Demographics (N = 15)

Characteristic	Participants, No. (%)
Race and ethnicity	
Asian	1 (6.7)
Black or African American	5 (33.3)
Hispanic	1 (6.7)
White	8 (53.3)
Gender	
Man	2 (13.3)
Woman	13 (86.7)
Age, mean (SD), y	44.7 (12.6)
Job role	
Social services director	8 (53.3)
Social workers or related role	3 (20.0)
Administrator or other role^a^	4 (26.7)

^a^ Included executive director, memory care coordinator, and behavioral unit manager of a locked unit.

### Barriers to Discharge

Barriers to discharge existed at the individual, relational, and structural levels. According to participants, residents with serious mental illness were reported to have difficulty transitioning from the nursing home given individuals’ behaviors and preferences; strained interpersonal and organizational relationships; and structural hurdles that leave no clear, safe pathways for successful discharge.

#### Individual Barriers

Nearly all participants told us that discharge planning starts at intake. While some participants said that they believed all residents would prefer to leave if given the choice, others noted that the residents themselves may be reluctant to leave the safe, structured environment provided by the facility (Table 3). As 1 staff member said:

We always ask when they’re admitted, “What’s your plan? What are we gonna do? Where did you live before you came here? Let’s set a goal. When do you think you want to go home?” We try to ask these things within their first 3 days because, trust me, if you don’t ask by day 3, by day 7, they’re comfortable, and they are like, “I’m not going anywhere. I’m staying right here. I came here with the intention to stay here.” (Participant 5)

Another participant noted:

**Table 3.** Individual Barriers to Discharge

Barrier	Example quotes
Resident reluctance to discharge	“When you make a referral to the [regional] homeless shelter system, the person has to consent to the placement. Now, if that person says, ‘No,’ then I can’t place them.” (Participant 15) “[Some patients] don’t want to tell us where they are going and that can make discharge planning harder.” (Participant 8) “Some of these people, when we try to help them, they aren’t willing to be helped. [We] get stuck with patients who refuse to leave.” (Participant 8)
Behavioral challenges of serious mental illness	“[Placement at group homes or other facilities] depends on the individual. It depends if [their behavior is] directed at staff. Is it just hallucinations? Is it just delusions? You know, are they hearing voices at one time of day, you know? Did they have any previous trauma that might be easily triggered? You need skilled staff to kind of deal with those behaviors.” (Participant 1) “If they have mental illness, more behaviors, they’re more than likely getting denied another placement.” (Participant 12)
Treatment adherence	“It really goes back to the medication management. Somebody who has a very serious mental health illness, they won’t always, by mouth, take their meds. So, what has to happen is, I have to make sure that by this…when they’re discharging from me, they have an avenue, that I know that they’re gonna at least take their meds.” (Participant 1) “It takes at least a month to [make] sure that a patient that is receiving those types of medications is stabilizing. because one thing with mental health is once they’re taking it, and they feel fine, they feel the need to not want to continue the medications because they think they’re fine. And yes, they’re fine because they’re on the medications, so they will stop. And then that’s when you can have those episodes of manic.” (Participant 6)

I’ve noticed with a lot of our residents that have schizophrenia or bipolar diagnosis, they have—I don’t want to say institutionalized—but they’ve become so accustomed to a routine that if you transition them into a community, it could set them back because they are used to a certain routine, if that makes sense. (Participant 1)

Participants observed that in addition to providing structure for residents, nursing homes may offer a sense of community that could be lacking in other settings. Recalling a resident who left but returned, 1 participant stated:

I think it’s really hard because, you know, when they’re in here, they’re, “When am I leaving? When am I leaving?” And then, when they leave, it’s like, “Oh, I’m by myself.” So that’s a rough [transition]. We had a lady who left and came back because she said she didn’t realize how lonely she was at home. (Participant 10)

Participants also emphasized that without sufficient preparation and planning, residents were likely to end up back in the hospital, on the streets, or in police custody. Furthermore, some residents with serious mental illness may experience hallucinations or delusions along with associated behaviors that are perceived as threatening, such as verbal outbursts or physical violence. When asked about what type of residents are particularly difficult to discharge, 1 participant said:

I would say, if there’s one that’s…behavioral. A lot of behaviorals involved…combative, verbally aggressive, or abusive. Maybe not connected with mental health [services], so that would be, I think, a barrier, a concern, an issue with placing. (Participant 6)

These challenging behaviors, if coupled with resistance to medication or therapy, make discharge planning even more difficult:

Unsuccessful discharge is [when] I’ve put out 10 referrals, and no one wants to take them. They’re having, just, behaviors. Every…week we’re doing med reviews….They’re refusing meds. Redirecting them is starting to get hard. Psychotherapy isn’t working; that’s triggering them for some odd reason. That’s the nightmare to it. At that point, there is no discharge. They’re…here. (Participant 1)

#### Relational Barriers

Ideally, when residents no longer require nursing home care, they would transition to their own home; that of a family member; or an alternative residential setting, such as a group home. However, these options require negotiation among the resident, those who may help with their care, and/or other organizations within the community. A commonly cited challenge for residents with serious mental illness was a dearth of supportive family relationships (Table 4). Some family members may not have the financial or emotional resources to offer support, or they may feel burned out from years of trying. One participant remarked:

**Table 4.** Relational Barriers to Discharge

Barrier	Example quotes
Absence of family support	“Sometimes the family [gets] comfortable with them being here; the family just wants to unload them. They’ll say, ‘I can’t take care of them anymore.’” (Participant 8) “A lot of [family members] just seem to not want to deal with the chaos of their loved one. Or they’ve just dealt with so much, they’ve just washed their hands of it.” (Participant 12) “A lot of [residents], especially with mental illness….They’re pretty much alone. And they might have 1 person, you know, a mom or sister [or] aunt, but that person is burnt out.” (Participant 10) “Yeah, honestly, with a lot of the [patients with serious mental illness], I would say they don’t typically have good support systems. They don’t really have, you know, family involved or friends involved, which, you know that that plays a big huge part in…getting them help or finding them help or resources they need to keep it going.” (Participant 7) “One thing I can say here for sure is there are a lot of family dynamics. So, you might have a resident that has a supportive cousin, but that cousin also does heroin. So, how stable and supportive is it to discharge them to their house? And a lot of times, what I’ve noticed is that those family members that just simply can’t support or don’t have the resources, they’re burnt out.” (Participant 1)
Limited understanding of serious mental illness in the community	“From the African American community, we don’t tend to do the psychiatrist and stuff like that and getting diagnosed. And we don’t feel like we need to take medicine and stuff like that. You just say, ‘Uncle such and such is senile,’ or ‘Cousin Rita is just, you know, she a little crazy.’ But the whole time, they have a diagnosis of bipolar and schizophrenia! But once it get[s] to the point where it’s uncontrollable, the family has cut all ties because they feel like that person know[s] what they’re doing and can help what they’re doing, when in reality they can’t.” (Participant 14)
Staff reluctance to potentially strain community relationships	“With serious mental illness you really just have to disclose everything because you don’t want to ruin the relationships that you have in the community. You always have to be straightforward with the placement because you don’t want it to ever be a surprise.” (Participant 5) “You can burn a lot of bridges with a very aggressive or noncompliant person. So then finding an appropriate place for them to go to can be extremely difficult because people don’t want to open their door to them.” (Participant 1)

There’s not a lot that have that support system, to be really honest. A lot of these patients have had substantial histories, or a lifetime of these [serious mental illnesses], and families are kind of at a point where they just don’t know how to help anymore. They are overwhelmed, overstimulated, and just kind of, for lack of better words, tapped out. (Participant 13)

Moreover, according to participants, some families or communities may not sufficiently understand serious mental illness and blame individuals for what they perceive as problematic behavior. For example:

Maybe [the family is] not even aware that that’s a diagnosis. You know, could be lack of knowledge or education. I don’t feel like there’s a lot of awareness with mental health and identifying different types of mental health [challenges]. (Participant 6)

In addition to the personal relationships of residents with serious mental illness, successful discharge is a function of relationships the nursing home has within the broader community, including group homes, assisted living communities, and shelters. Participants reported that they risked damaging relationships with community partners if they referred a resident with serious mental illness who exhibited potentially distressing behaviors, such as verbal or physical outbursts. In the interest of maintaining long-term relationships with community partners, they were reluctant to refer a resident with potentially disruptive behavior or other symptoms.

#### Structural Barriers

Finally, structural barriers, specifically access to services and financial and regulatory constraints, limit discharge (Table 5). One residential option might be able to accommodate mental illness but not a comorbid substance use disorder; another might have age requirements; or the seemingly perfect fit could have a 6-month waiting list. One participant described the misalignment between a resident’s needs and available community options:

**Table 5.** Structural Barriers to Discharge

Barrier	Example quotes
Access issues	
Barriers to postdischarge settings (eg, based on age, abilities, comorbidities)	“If we have someone that’s homeless and comes in with an addiction problem, and they want treatment, as soon as they’re medically stable, I can send them to [a] drug and alcohol program, like inpatient. But you have to be medically okay before they will take you. So anybody else that comes in with that and is homeless are just gonna end up staying here until we can find somewhere for them to discharge to in the community, like housing, or whatever, and go through the programs on their own.” (Participant 4) “They don’t have any family. They don’t have the financial means to make it on their own, even if they are capable. It’s just hard to find resources, for, I mean, there is low-income housing, but there’s wait lists. There’s a lot of stipulations. We get a lot of younger people, you know, in their late 40s, early 50s who would be great for assisted living. But they’re too young.” (Participant 10) “The age is a big barrier. There’s not a lot of places for younger people. If I have a client that’s having behaviors that need to go out and get what we call the ‘med adjustment,’ it’s hard to find placement close in…proximity. It’s like, you have to go further out. Around here, it’s like 62 and up. So 62 on down, it’s hard. It’s very hard.” (Participant 14)
Waiting lists	“It’s difficult here. Here [in this rural region] especially, we only have 2, 3 local providers that have psychiatric doctors and therapists…the wait list was 4 months.” (Participant 4) “Senior apartments are all full. The waiting list is 3 years long.” (Participant 1)
Transportation	“Transportation, that is a huge barrier. Huge. That’s probably the second biggest barrier that we have. We can connect you to all these resources, but how are you going to get there?” (Participant 1)
Nowhere else to go	“[Residents with serious mental illness] literally have been through this process multiple times prior to getting here. So there’s literally nowhere else to go. There aren’t any inpatient psych[iatric] facilities, where they really need to be, where they can get the treatment that they really need. So we’re kind of a surrogate.” (Participant 5) “A lot of the time, it seems like hospitals are dumping patients on us just to get them out of the hospital.” (Participant 8) “Honestly, most of the patients that we see here, they have spent years in long-term-care facilities, and they’ve gone from facility to facility and had unsuccessful stays, been kicked out from those facilities. And then we’re kind of like that spot that collects those, you know, keeps them and loves them and gives them a sense of, ‘I’m home,’ here.” (Participant 14)
Financial or regulatory barriers	“Finding an appropriate place for them to go to can be extremely difficult because they might not meet the criteria for [an] inpatient facility either. So there’s just not a lot [as] far as housing for someone with a serious mental health issues. And then the financial aspect of it because someone with a mental health disorder isn’t gonna be making a lot of money. A lot of them don’t have a huge monthly income where, you know, they could afford the luxuries of a really nice group home.” (Participant 1) “I wish that there was like a big building that I could just discharge them all to and know that they were getting taken care of all the time and that we, like, were able to go check on them. A place that they didn’t require income to get help.” (Participant 5) “[Home health] is not there long enough to provide the amount of care, especially right after a discharge, to a resident in their home. It’s a very scary transition when you go from 24-hour care, and then you’re in your home and they come out, they visit, they’re there for a short time, and it’s like, ‘Okay, we’ll see you in a couple of days,’ and it’s a couple of times a week. And then they’re left on their own.” (Participant 6) “If someone ends up staying within a longer time, like more than 90 days, then what happens is they would, like, lose their bed at that [alternative] facility, and then we and then they have to get approval by the department of health to get readmitted because there was a class action lawsuit a number of years ago in which there were people who were saying that adult living facilities were warehousing people with serious mental illness, you know, and not providing with the appropriate care. Anyhow, the lawsuit was won, so now adult homes can’t have more than 25% capacity with [serious mental illness].” (Participant 15)

Let’s say someone’s got a serious mental health diagnosis, but they also are in a wheelchair. So, I’ve got, like, what I call a double-whammy there, you know what I’m saying? Those are the ones that take the longest because they aren’t independent enough to bathe themselves or see themselves, or they can’t get upstairs. (Participant 1)

Participants commented on the revolving doors between systems of care, including hospitals, nursing homes, and alternative care options, all exacerbated by waiting lists for mental health services. One participant said:

[I]f they don’t have somebody prescribing meds, they go into crisis again. It’s just like a vicious cycle, like they’re just coming back. They’re going back to the hospital because it’s not available to them. It’s ridiculous how long you have to wait as a new patient. (Participant 4)

In rural regions, skilled psychiatric resources were especially hard to come by, with months-long waiting lists. Even when an appointment was available, potential patients could not get to these clinicians without a reliable car or public transportation. One participant said:

In [the city a couple hours away], there’s a program for everything. We have nothing like that. Like, we have some groups for, like, drug and alcohol up here, very few. We struggle with transport. There’s not enough mental health providers in this area at all. If you don’t drive…we don’t have a lot. (Participant 4)

In less rural areas, psychiatric support might be more readily accessible, but affordable housing could have a waiting list 3 years long and be on the other side of the city, far removed from a patient’s neighborhood and limited support network.

In the rare event that a resident could return to stable housing, they may not be able to receive the in-home support necessary to live safely. One participant shared:

I think once they go out, [home health care is] maybe 2 visits a week, and probably 15 to 30 minute[s]…of time [is] spent with the resident. It’s not very long and not enough time [that] residents need. (Participant 6)

Finally, payment was also a commonly cited barrier. Finding a post–nursing home placement that could meet all of a resident’s care needs, had an available spot, and would be covered by insurance was a challenge. If a placement was a good fit but had a waiting list, insurers might not be willing to pay for the resident’s continued care in the nursing home setting while they waited for a spot to open. As 1 participant described:

If I put them on a wait list, then their insurance might not pay for them to be here that long, and until that wait list is up. Then it comes down to, if they stay here, then they can’t have any financial savings. If you’re on Medicaid, which is normally what long-term residents are on, or somebody who is here for 6 months to a year…you can’t have more than $2000 in your account. And then once you become a long-term resident, or you want to be here for, you know, 6 months to a year, they take pretty much all your money, and you get $52 a month. (Participant 10)

## Discussion

This qualitative study of nursing home staff across the US provides novel insights into the challenges of discharging residents with serious mental illness, an important extension of the prior qualitative work focused on the challenges of delivering appropriate care in this setting.^17,18^ In the decades following the Olmstead decision, the proportion of nursing home residents with serious mental illness has increased markedly, reflecting the reality that nursing homes are the “de facto destination for individuals with mental illness” in the US.^19^^(p628)^ Our findings also support previous quantitative work showing that patients with serious mental illness may be at higher risk of becoming long-stay nursing home residents despite having fewer functional needs,^7^ which may be a function of barriers to discharge described here. Staff who participated in this study conveyed a range of barriers, including behavioral symptoms, lack of support networks and stable housing, and a dearth of placement options able to accept individuals with serious mental illness who may also be experiencing other comorbidities.

Nursing home staff must work with residents, and ideally, their broader support networks, on a safe discharge plan. Discharge may be to any number of settings based on care needs, ranging from assisted living facilities to living alone or with relatives in a house or apartment, and typically includes arranging any follow-up outpatient appointments. Discharge planning may stall without an appropriate residential setting, though that is only part of the unique psychiatric, physiologic, and psychosocial needs of individuals with serious mental illness. Families of residents with serious mental illness are often depleted financially or emotionally and may be less willing or able to participate in discharge planning. Lack of family support during discharge is associated with increased psychiatric symptoms and hospitalization and decreased use of follow-up outpatient psychiatric services for individuals with serious mental illness.^20,21^ Unreliable access to transportation can lead to missed appointments and lower prescription fill rates,^22^ and for individuals with serious mental illness, this could result in difficulty adhering to their medication regimen, exacerbating psychiatric symptoms, and increasing their risk of hospitalization.^23^ Ultimately, nursing home staff may encounter unique challenges and numerous overlapping barriers throughout the discharge planning process for individuals with serious mental illness.

The premise behind closing public psychiatric facilities (ie, deinstitutionalization) during the 1950s was that previously hospitalized individuals would be supported and able to live independently in community settings.^2,7^ However, without adequate investments in community supports (eg, home- and community-based services [HCBSs]), individuals with mental illness simply shifted to alternative institutional settings such as nursing homes.^9,19^ As such, nursing homes located in areas with a higher ratio of HCBS recipients to nursing home residents have higher community discharge rates,^24^ and HCBS spending has been posited as a reason for large state-level variation in the nursing home population with serious mental illness.^25^ However, HCBS spending and higher discharge rates overall may not translate to higher discharge rates specifically for residents with serious mental illness. While some potential nursing home residents may benefit from investment in HCBS, such services alone are inadequate if, for example, there are no stable housing options.

Addressing the intersecting individual, relational, and structural barriers to discharge may require a concerted and comprehensive approach that considers these multiple levels of obstacles and the needs of individuals with serious mental illness across settings. This approach may include offering education and training for staff and families to address behaviors and stigma of serious mental illness^26^; adequately funding community mental health services^27,28^; enhancing a community’s transportation infrastructure^29^; and expanding tiered supportive housing options, such as assisted living facilities with embedded mental health and/or substance use support or group homes integrated with the community mental health system. In addition, long-acting injectable antipsychotics offer substantial benefits over oral formulations and may be beneficial to offer individuals who are approaching discharge,^30,31^ though to our knowledge, there are no data on their use in the nursing home setting.

### Limitations

This study has some limitations. We began with 74 potential facilities and ultimately identified only 8 willing to participate. While these facilities represent a national sample that varied on key facility characteristics and we achieved saturation during analysis, our findings may not broadly generalize across nursing homes nationally. Furthermore, interviews were with the subset of nursing home staff most responsible for discharge planning. Future work should involve other clinical roles, the residents themselves, and family members or friends. Finally, to explore structural barriers, future work needs to consider regional differences in policy and resources.

## Conclusions

This qualitative study of nursing home staff found multiple challenges at the individual, relational, and structural levels associated with the safe and timely discharge of residents with serious mental illness. Our findings suggest the need to develop collaborative community strategies and policies that efficiently allocate resources and address these challenges to ensure safe, supportive, and sustainable options for individuals with serious mental illness once they leave the nursing home.

## Notes

Supplement 1. **eMethods.** Barriers to Discharge Nursing Home Residents With Serious Mental Illness Interview Guide

Supplement 2.
Data Sharing Statement
