# Supplementary material for: Barriers to Discharge for Nursing Home Residents With Serious Mental Illness
Source: JAMA Netw Open. 2025 Sep 30;8(9):e2534685. doi: 10.1001/jamanetworkopen.2025.34685 (PMC12485640; doi:10.1001/jamanetworkopen.2025.34685)
Supplement: Supplement 1. — eMethods. Barriers to Discharge Nursing Home Residents With Serious Mental Illness Interview Guide [file jamanetwopen-e2534685-s001.pdf]

Supplemental Online Content

Hoffman CM, Ratliff HC, Krein SL, et al. Barriers to discharge for nursing home residents with serious mental illness. *JAMA Netw Open*. 2025;8(9):e2534685.
doi:10.1001/jamanetworkopen.2025.34685

eMethods. Barriers to Discharge Nursing Home Residents With Serious Mental Illness Interview Guide

This supplemental material has been provided by the authors to give readers additional information about their work.

© 2025 Hoffman CM et al. *JAMA Network Open*.

eMethods. Barriers to Discharge Nursing Home Residents With Serious Mental Illness Interview Guide

Introduction

- Introduce who is present and what their role will be
- Explain the purpose of the interview
- Ask if there are any questions
- Tell them when the recorders are turned on

Background Information

To begin, we have a few questions about your background.

1. What is your role at your facility?
2. How long have you been in your current position?

For the rest of our conversation, we would like to focus specifically on the care of individuals with serious mental illness at your facility. By serious mental illness we are specifically referring to schizophrenia, other psychotic disorders, or bipolar disorder. We are not referring to depression or anxiety.

We are particularly interested in learning about what facilitates a successful discharge—meaning back to a community setting—for individuals with SMI.

3. Could we start with a high-level overview of what discharge planning for patients in your facility looks like?
    Probe – who is usually involved, both within the nursing home and outside of it?
4. Compared to facility residents <u>without</u> serious mental illness, are there unique or additional elements for discharge planning for individuals with SMI?
    a. *If yes, elaborate?*
5. I am sure you work with many residents with dementia. How does the process for discharging someone with dementia look vs. someone with serious mental illness?
6. Are there particular barriers to discharge planning for individuals with serious mental illness? Could you elaborate? *Probe for . . .*
    a. Difficulties related to arranging housing?
    b. Difficulties related to arranging psychiatric treatment?
    c. Difficulties related to arranging non-psychiatric medical care?
    d. How do these compare relative to barriers other patients face?
7. When discharge planning for individuals with SMI, how often are family members or partners involved?
    a. Is this similar to your residents without SMI?
    NOTE for interviewer: this may have already been addressed in the answers to #6
8. Are there particular types of SMI patients for whom a successful discharge is especially hard to facilitate?
    a. *If yes, elaborate?*
9. Are there particular resources or services in the community that help facilitate a successful discharge for these individuals?
10. Do you find that individuals with SMI would prefer to remain a resident of your facility as opposed to being discharged to the community?

© 2025 Hoffman CM et al. *JAMA Network Open*.

11. If you could make one single change that would help you improve your ability to discharge patients with SMI to the community, what would that change be?
12. Can you think of a successful example of discharging a person with serious mental illness to the community? What made it a success? How about a time when it did not go well?
13. We would like to end with a few demographic questions:
    a. Age
    b. Gender
    c. Race
    d. terminal degree

© 2025 Hoffman CM et al. *JAMA Network Open*.
