# Supplementary material for: Barriers to Discharge for Nursing Home Residents With Serious Mental Illness
Source: JAMA Netw Open. 2025 Sep 30;8(9):e2534685. doi: 10.1001/jamanetworkopen.2025.34685 (PMC12485640; doi:10.1001/jamanetworkopen.2025.34685)
Supplement: Supplement 2. — Data Sharing Statement [file jamanetwopen-e2534685-s002.pdf]

# Data Sharing Statement

Hoffman. Barriers to Discharge for Nursing Home Residents With Serious Mental Illness. *JAMA Netw Open*. Published September 30, 2025. doi:10.1001/jamanetworkopen.2025.34685

**Data**
**Data available:** Yes
**Data types:** Deidentified participant data
**How to access data:** www.icpsr.umich.edu
**When available:** beginning date: 08-31-2026

**Supporting Documents**
**Document types:** None

**Additional Information**
**Who can access the data:** Anyone requesting the data.
**Types of analyses:** For any purpose.
**Mechanisms of data availability:** Data is accessible via ICPSR to anyone who wishes to access and download.
